# Assessment of Purple Loosestrife (*Lythrum salicaria* L.) Extracts from Wild Flora of Transylvania: Phenolic Profile, Antioxidant Activity, In Vivo Toxicity, and Gene Expression Variegation Studies

**DOI:** 10.3390/pharmaceutics17091097

**Published:** 2025-08-22

**Authors:** Lidia-Ioana Virchea, Cecilia Georgescu, Endre Máthé, Adina Frum, Monica Mironescu, Bence Pecsenye, Robert Nagy, Oana Danci, Maria-Lucia Mureșan, Maria Totan, Felicia-Gabriela Gligor

**Affiliations:** 1 Faculty of Medicine, “Lucian Blaga” University of Sibiu, Lucian Blaga Str. 2A, 550169 Sibiu, Romania; lidia.virchea@ulbsibiu.ro (L.-I.V.); adina.frum@ulbsibiu.ro (A.F.); maria.muresan@ulbsibiu.ro (M.-L.M.); maria.totan@ulbsibiu.ro (M.T.); felicia.gligor@ulbsibiu.ro (F.-G.G.); 2 Faculty of Agriculture Sciences, Food Industry and Environmental Protection, “Lucian Blaga” University of Sibiu, Dr. Ion Rațiu Str. 7-9, 550012 Sibiu, Romania; monica.mironescu@ulbsibiu.ro; 3 Institute of Nutrition Science, Faculty of Agricultural and Food Sciences and Environmental Management, University of Debrecen, Böszörményi Str. 128, 4032 Debrecen, Hungary; pecsenye.bence@agr.unideb.hu (B.P.); nagy.robert@agr.unideb.hu (R.N.); 4 Department of Life Sciences, Faculty of Medicine, Vasile Goldis, Western University from Arad, L. Rebreanu Str. 86, 310414 Arad, Romania; 5 Faculty of Sciences, “Lucian Blaga” University of Sibiu, Dr. Ion Rațiu Str. 5-7, 550012 Sibiu, Romania; oana.danci@ulbsibiu.ro

**Keywords:** *L. salicaria* L., phenolic compounds, antioxidant, toxicity, gene expression

## Abstract

**Background:** Purple loosestrife (*Lythrum salicaria* L.) is a medicinal plant native to the spontaneous Romanian flora. The aim of this study was to investigate the phenolic profile, total phenolic content (TPC), and antioxidant capacity (AC) of two *L. salicaria* L. extracts, a hydro-methanolic extract (LSmet-1) and a hydro-ethanolic extract (LSeth-2), and their putative toxicity, as well as the effect on eye pigment content in the case of *Drosophila melanogaster* of an extract derived from LSmet-1 (LSmet-3). To the best of our knowledge, this is the first study to evaluate the influence of *L. salicaria* L. extracts on cytotoxicity and the expression of genes as determined by eye pigment levels, using a *D. melanogaster*-based model system. **Methods:** High-performance liquid chromatography was carried out to investigate the chemical composition of the extracts. Spectrophotometric methods were used to estimate their TPC and AC. Cytotoxicity was evaluated using an in vivo *D. melanogaster* diet-dependent viability assay and eye pigments of *w^m4h^* males, suitable for position-effect variegation studies, which were quantified by a spectrophotometric method. **Results:** The results indicated that the main phenolic compounds were gallic acid, resveratrol, and rutin in LSmet-1, whereas in LSeth-2, gallic acid and quercetin were the most relevant. LSmet-1 had a higher TPC compared to LSeth-2. Both extracts exhibited notable efficacy in the applied in vitro antioxidant tests. The viability of flies on normal media increased in a concentration-dependent manner at lower concentrations, with the extract being toxic at higher concentrations. On a high-sugar diet, even lower concentrations were toxic. All tested concentrations influenced the eye pigment content. **Conclusions:** Our study brings new findings on *L. salicaria* L. extracts, suggesting the need for further investigation before introducing them in therapy.

## 1. Introduction

Medicinal plants are valuable natural sources of phytocompounds with biological properties [1]. *Lythrum salicaria* L. is a medicinal plant species belonging to the *Lythraceae* family, known by its common name purple loosestrife [2]. Furthermore, it is an invasive species that can be exploited by the pharmaceutical industry for the development of new and sustainable products [3]. *Lythri herba*, dried flowering tops, is officialized in the European Pharmacopoeia 10th edition [4]. *L. salicaria* L. has been used in traditional medicine to alleviate gastrointestinal tract disorders such as diarrhea and dysentery, hemorrhages, and skin and mucosal disorders [2]. Several studies have assessed the chemical composition [3,5,6,7] and biological activities including antioxidant [3,6,8,9,10,11,12,13,14], antimicrobial [3,6,9,15,16], anti-inflammatory [8,14,17], anti-nociceptive [8] and wound-healing properties [18], protection and stimulation of skin cells [19], ileum smooth muscle contraction [5], anti-obesity [20] and weak anti-diabetic [10] effects, and toxicity [12,21] of *L. salicaria* L. extracts. Additionally, polysaccharide–polyphenol conjugates isolated from this plant were reported to possess antitussive and bronchodilator properties [22], as well as controversial anticoagulant and pro-coagulant effects [23].

Polyphenols are considered a broad class of secondary plant metabolite and are typically divided into phenolic acids, flavonoids, tannins, lignans, and stilbenes, based on their chemical structures. About 8000 polyphenols are currently known [24]. *L. salicaria* L. extracts were reported to contain high amounts of phenolic acids including caffeic [5], chlorogenic [5,25], isochlorogenic [25], ellagic, and gallic acids [3,5,25], as well as flavonoids such as apigenin, catechin, quercetin-3-D-galactoside, luteolin [5], orientin [3,5,9,20,25], isoorientin [3,5,8,9,25], rutin [5,9], vitexin [3,5,9,25], and isovitexin [5,8,25]. Ellagitannins such as castalagin [19,25,26,27,28], pedunculagin [25], vescalagin [19,25,26,27,28], and salicarinins (A, B [26,27,28], and C [27,28]) are another important group of compounds from *L. salicaria* L. extracts. In addition, other researchers have isolated triterpenes and coumarins [29].

Polyphenols possess numerous biological activities, such as antioxidant, antimicrobial, anti-inflammatory, anti-diabetic, cardioprotective, and anticancer effects [24,30]. The antioxidant effect of polyphenols is attributed to their ability to scavenge reactive oxygen species (ROS) of endogenous or exogenous origin. When ROS exceeded the body’s defense capacity, oxidative stress occurs, leading to protein, lipid, carbohydrate, and nucleic acid damage, which involves the development of chronic diseases including inflammation, diabetes, cardiovascular diseases, and cancer [31]. *L. salicaria* L. extracts demonstrated antioxidant activity by scavenging 2,2-diphenyl-1-picrylhydrazyl (DPPH) and 2,2′-azinobis [3-ethylbenzothiazoline-6-sulfonic acid]-diammonium salt (ABTS) free radicals. Regarding the bioactive compounds, gallic acid showed the strongest activity against DPPH free radicals, followed by ellagic and caffeic acid [3]. The incorporation of *L. salicaria* L. extract into sodium alginate films exerted a concentration-dependent antioxidant effect against both DPPH and ABTS radicals [32].

In the context of the extensive use of plant-based products in therapy, their toxicity investigation is crucial [33]. *Drosophila melanogaster* (fruit fly) is one of the most widely used model organisms in biological research, especially for investigating the developmental toxicity induced by plants and plant derivatives [34]. *Drosophila melanogaster* is a versatile organism due to its features including inexpensiveness, small size, ease of manipulation, short life cycle, and approximately 75% similarity between its disease-related genes and those of humans, as well as its fewer ethical and safety issues compared to vertebrates [35]. The ability to easily observe changes induced by natural products through dietary exposure makes *Drosophila melanogaster* a promising organism for medicinal plant research [34].

In this background, the aim of our study was to investigate two extracts of *L. salicaria* L. collected from mountainous regions considered unpolluted regarding their phenolic profile, total phenolic content, and antioxidant capacity and assess the toxicity and the influence on eye pigment content of different extract concentrations in a *Drosophila melanogaster* model system. To our knowledge, no previous studies have examined the impact of *Lythrum salicaria* L. extracts on the toxicity and eye pigment content of *Drosophila melanogaster*.

## 2. Materials and Methods

### 2.1. Chemicals

Methanol, glycerin, acetic acid, DPPH, 2,4,6-tripyridyl-s-triazine (TPTZ), chloroform, and ammonium hydroxide were purchased from Sigma-Aldrich Company, St. Louis, Missouri, USA. (+)-Catechin, cinnamic acid, caffeic acid, chlorogenic acid, ferulic acid, gallic acid, syringic acid, resveratrol, rutin, and quercetin standards were obtained from Dr. Ehrenstorfer GmbH, Augsburg, Germany. Phosphomolybdotungstic reagent (Folin–Ciocâlteu’s reagent), potassium persulfate, charcoal, agar powder, and saccharose were acquired from VWR Chemicals, Leuven, Belgium. Sodium carbonate, sodium acetate, ferric chloride, hydrochloric acid, and ascorbic acid were purchased from Chemical Company, Iași, Romania. Trolox, ABTS and nipagin were obtained from Thermo Fisher Scientific, Kandel, Germany. Ethanol was acquired from Chimreactiv S.R.L., Bucharest, Romania.

### 2.2. Plant Collection and Preparation

*L. salicaria* L. flowering aerial parts were collected from Sibiu County, Romania (45°41′20″ N, 23°59′19″ E, 1100 m altitude), in July 2021. The plant was authenticated by the botanist Dr. Oana Danci using the *Vascular Plants of Romania, illustrated field guide* by Sîrbu et al. (2013) [36]. The sample voucher specimen (no. 101/1) is held at the “Lucian Blaga” University of Sibiu, Romania, in the Faculty of Medicine, pharmacy specialization. The vegetal raw material was kept in the shade at room temperature until drying, and then it was chopped and stored in paper bags at room temperature, protected from light and humidity, until extraction.

### 2.3. Bioactive Compound Extraction

The dried plant material was milled using a domestic electric grinder, until a powder was obtained. Then, two types of extracts were prepared. For the first extract (LSmet-1), 0.5 g of ground plant was mixed with 10 mL of solvent mixture (methanol/distilled water, 70:30, *v*/*v*) in an Erlenmeyer flask with a ground-glass stopper. The extraction was carried out in an ultrasonic water bath at 40 °C for 30 min. After cooling, the mixture was filtered into a 10 mL volumetric flask and the filtrate was brought to 10 mL with the same solvent [37]. The 70:30 methanol/water ratio was chosen based on a previous study conducted by Frum et al. (2018), which reported higher phenolic compound recovery by using this solvent mixture compared to methanol alone [38]. For the second extract (LSeth-2), the same method was applied, except using 1 g of ground plant and replacing methanol with ethanol. The TPC, phenolic profile, and antioxidant activity assays were conducted immediately after the extractions were performed as mentioned before. For the tests conducted on *Drosophila melanogaster*, the methanol toxicity was avoided by evaporating it from LSmet-1 (because a higher polyphenol content and antioxidant activity was expected for the hydro-methanolic extract). Subsequently, the resulting product was mixed with glycerin in a 2:1 *v*/*v* ratio, thus obtaining the final mixture (LSmet-3).

### 2.4. Phenolic Profile HPLC Analysis

Phenolic compounds were identified and quantified in the extracts by HPLC-UV, using the SCL-40 HPLC apparatus (Shimadzu, Kyoto, Japan). The method was adapted from previously described protocols [37,39]. The system was equipped with a degasser, quaternary pump, photodiode array detector, thermostatted column oven, and autosampler. A Nucleosil C18 column (250 mm × 4.6 mm, i.d. 5 µm) set to 40 °C was used. The mobile phases consisted of two components: (A) a mixture of purified water and acetic acid in a ratio of 96:4, *v*/*v* and (B) methanol. The elution was conducted in gradient, following this program: at 0 min—85% (A) and 15% (B), at 15 min—75% (A) and 25% (B), at 20 min—15% (A) and 85% (B), at 30 min—15% (A) and 85% (B), at 35 min—85% (A) and 15% (B), and at 36 min— 85% (A) and 15% (B). A volume of 5 μL of each sample was injected. The flow rate was 0.8 mL/min. Ten standards for phenolic compounds were used and the detection was performed at the following wavelengths at specific retention times (Rt): gallic acid (Rt~5.1 min), (+)-catechin (Rt~8.8 min), syringic acid (Rt~16.9 min) and cinnamic acid (Rt~24.2 min) at 280 nm; resveratrol (Rt~23.0 min) at 306 nm; chlorogenic acid (Rt~12.5 min), caffeic acid (Rt~14.5 min), and ferulic acid (Rt~22.1 min) at 330 nm; and rutin (Rt~22.8 min) and quercetin (Rt~24.4 min) at 360 nm (Figures S1–S3). The tested concentration range was 0.2–50 μg/mL, and the regression equations are provided in the Supplementary Materials, Table S1. The results were expressed as μg of compound/g of dry plant.

### 2.5. Spectrophotometric Analysis

#### 2.5.1. Total Polyphenol Content Assay

To determine the TPC of the extracts, the Folin–Ciocâlteu method was adapted from Frum et al. (2022). A volume of 0.4 mL of extract, 1 mL of Folin–Ciocâlteu reagent, 10 mL of distilled water, and 2 mL of 290 g/L sodium carbonate solution were mixed in test tubes. After 10 min of shaking, the tubes were incubated into a water bath at 40 °C for 20 min. After cooling at room temperature, the absorbance was measured at 760 nm against a blank by using a Shimadzu UV 1900 spectrophotometer (Shimadzu, Kyoto, Japan) [40].

A calibration curve with concentrations between 0.86 and 8.57 μg gallic acid/mL was plotted. Triplicate analyses were performed for each sample. The results were expressed as mg gallic acid equivalents (GAE)/g dry plant.

#### 2.5.2. Assessment of Antioxidant Capacity

##### DPPH Assay

To investigate the DPPH scavenging activity of the extracts, a method adapted from Georgescu et al. (2022) was conducted. A 25 μg/mL DPPH stock solution was prepared and left in the dark for 2 h before analysis. In a test tube, a volume of 1940 μL of DPPH stock solution was mixed with 60 μL of extract and kept in a dark place for 15 min. The absorbance was measured at 515 nm against a blank by using a Shimadzu UV 1900 spectrophotometer (Shimadzu, Kyoto, Japan) [39]. Ascorbic acid was used as a positive control.

A calibration curve with concentrations between 0.5 and 25 μg DPPH/mL was plotted. Triplicate analyses were performed for each sample.

The concentration of DPPH in the sample was calculated using Formula (1):

(1) $$C_{sample}=\frac{A_{sample\;}-\;i}{s}$$

where $C_{sample}$ = the concentration of the DPPH in the sample solution in μg/mL; i = the intercept of the calibration curve; and s = the slope of the calibration curve.

The percentage of DPPH free radical scavenging activity (%RSA) was calculated using Formula (2):

(2) $$\%\mathrm{RSA}\;=\;\frac{C_{stock}\;-\;C_{sample}}{C_{stock}}*100$$

where $C_{stok}$ = the concentration of the DPPH stock solution in μg/mL and $C_{sample}$ = the concentration of the DPPH in the sample solution in μg/mL.

##### FRAP Assay

The reducing power of the extracts, which is correlated with their antioxidant activity, was evaluated by the FRAP assay, using a method adapted from Vicaș et al. (2015). The following stock solutions were prepared: 300 mM acetate buffer (pH = 3.6), 20 mM FeCl_3_, and 10 mM TPTZ acidified with 150 μL HCl. The FRAP solution was freshly prepared by mixing acetate buffer, FeCl_3_, and TPTZ solutions mixed in a volume ratio of 10:1:1. For the analysis, 100 μL of plant extract was mixed in a test tube with 0.5 mL of FRAP solution and 2 mL of distilled water. The test tube was kept in the dark for 1 h, after which the extinction was measured at 595 nm against the blank using a Shimadzu UV 1900 spectrophotometer (Shimadzu, Kyoto, Japan) [41]. Ascorbic acid was used as a positive control.

A calibration curve with concentrations between 0.15 and 0.50 μmol trolox/mL was plotted. Triplicate analyses were performed for each sample. The results were expressed as μmol trolox equivalent (TE)/g dry plant.

##### ABTS Assay

To test the antioxidant activity of the extracts using the ABTS assay, a method adapted from Vicaș et al. (2015) was applied. Sixteen hours before analysis, a 2.45 mM potassium persulfate and 7 mM ABTS stock solution was prepared and kept in a dark place. After this period, the solution was diluted until an extinction of 0.70 ± 0.02 at 734 nm was reached. For the analysis, 25 μL of each plant extract was mixed with 2.5 mL of the diluted ABTS solution and then vortexed for 30 s to ensure proper mixing. Next, the extinction was measured at 734 nm against a blank by using a Shimadzu UV 1900 spectrophotometer (Shimadzu, Kyoto, Japan) [41]. Ascorbic acid was used as a positive control.

A calibration curve with concentrations between 0.06 and 2.09 mmol trolox/L was plotted. Triplicate analyses were performed for each sample. The results were expressed as mmol TE/g dry plant.

### 2.6. In Vivo Drosophila melanogaster Viability Tests

To study the in vivo effect of LSmet-3 extract on the viability of *Drosophila melanogaster*, an assay adapted from Aleya et al. (2023) was conducted [42]. We prepared three types of culture medium: (1) neutral, (2) normal, and (3) high-sugar types. The neutral medium contained no nutrients and did not support larval development. Consequently, embryos that completed metamorphosis hatched as first-stage larvae but died shortly thereafter due to nutrient deficiencies, hence the zero-diet type of name. The zero-diet type of media was prepared by mixing 1 g of charcoal, 1 g of agar powder, and 100 mL of distilled water. The distilled water was previously boiled to ensure sterility, after which the other ingredients were added successively, followed by boiling the mixture for 30 s with continuous stirring. The mixture was then cooled to 45 °C and dispensed into culture vials.

The normal medium simulated a normal diet containing all necessary nutrients and was prepared as follows: in 1200 mL of distilled water, 70 g of yeast paste was added and stirred until homogeneous. In this mixture, 52 g of saccharose and 30 g of wheat flour were introduced and brought to a boil under continuous stirring, followed by the addition of 10 g of agar powder. After 30 min of boiling under continuous stirring, the mixture was cooled at 50 °C. Finally, 1 g of nipagin was dissolved to prevent mold contamination. The high-sugar medium was prepared similarly to the normal medium, except that a 0.75 M concentration of sucrose was used [42]. Normal and high-sugar media support the normal and high-sugar types of diets, respectively.

Three concentrations of the LSmet-3 prepared as given in Section 2.3 were tested on both the neutral and high-sugar media at concentrations like 4.76%, 20%, and 33.33%. These concentrations were prepared by adding 100 μL, 500 μL, and 1 mL of extract, respectively, to 2 mL of media in the test vials. Additionally, the extract concentration of 50%, obtained by mixing 2 mL of extract with 2 mL of neutral medium, was tested at neutral diet. Furthermore, seven concentrations of LSmet-3 were tested in the normal medium: 0.5%, 1.23%, 2.44%, 4.76%, 20%, 33.33%, and 50%. They were obtained by adding 10 μL, 25 μL, 50 μL, 100 μL, 500 μL, 1 mL, and 2 mL of LSmet-3 extract to normal media in the test vials. All vials were homogenized by shaking and left overnight to allow the media to solidify. The extract concentrations were chosen based on the study conducted by Aleya et al. (2023) that investigated the viability of plant extracts on *Drosophila melanogaster* with extract concentrations ranging from 11 to 50% [42]. Based on our observations in a neutral medium, additional extract concentrations ranging from 0.5 to 50% were tested in a normal medium. It was expected that this would provide a better understanding of the relationship between plant extract concentration and fly viability.

The tested *Drosophila melanogaster w^m4h^* embryos were obtained by placing approximately 50 males and 50 females into an egg collector positioned above a Petri dish filled with neutral medium. An optimal amount of yeast paste was placed in the middle of the dish. The Petri dish was replaced every two hours, and therefore, synchronized 0–2-hour-old embryos could be collected to set the viability experiments. All the experiments were conducted at 25 °C under controlled humidity conditions. After the media had solidified, 50 *w^m4h^* embryos were transferred into each test vial and their life cycle monitored. The newly formed pupae and hatched flies were counted daily. Triplicate analyses were performed for each sample. The results were expressed as a percentage of viability [42].

### 2.7. Drosophila melanogaster Eye Pigment Quantification

To quantify eye pigments of *Drosophila melanogaster w^m4h^*, a method adapted from Evans and Howells (1978) was used [43]. This experiment was conducted on flies raised on normal medium enriched with LSmet-3. Adult flies obtained from each extract concentration were transferred to separate vials containing normal media. Ten adult males from each group were randomly selected, and their heads were separated from the bodies under a VWR VisiScope SZB260 stereo microscope (VWR, Milano, Italy) using a scalpel. For each concentration, ten heads were mixed with 300 μL of CHCl_3_ and 300 μL of 0.1% NH_4_OH. After homogenization, the samples were centrifuged for 4 min at 4000 revolutions per minute. Next, 200 μL of the supernatant was mixed with 500 μL of 0.1% NH_4_OH. Following another round of homogenization, extinctions were measured at 485 nm against ethanol by using a UV/Vis Ultrospec 2100 pro spectrophotometer (Biochrom Ltd., Cambridge, England).

### 2.8. Statistical Analysis

The results are expressed as the mean and standard deviation (SD) of three replicates. An independent sample t-test and one-way ANOVA (Tukey’s post hoc test) were performed to assess the significance of differences between means. All statistical tests were computed using IMB SPSS statistics for Windows, version 26 (IBM Corp., Armonk, NY, USA).

## 3. Results and Discussions

### 3.1. Phenolic Profile

As presented in Table 1, seven compounds were identified and quantified in both extracts. Gallic acid, resveratrol, and rutin were the most abundant in the LSmet-1 hydro-methanolic extract, while quercetin and gallic acid were the main components in the LSeth-2 hydro-ethanolic extract. The quantities of each polyphenol are different among the two extracts. However, the biggest difference was observed in the content of quercetin, which was about 7-fold higher in the LSeth-2 extract compared to LSmet-1. The content of gallic acid was just about 1.36-fold higher in LSmet-1 compared to LSeth-2. Resveratrol and rutin were more abundant in LSmet-1 than in LSeth-2, with about 5- and 3-fold, respectively. Statistical analysis showed that the hydro-methanolic extract contains significantly higher amounts of gallic acid, rutin, and resveratrol than the hydro-ethanolic extract. However, the latter contains a significantly higher quercetin content than the former.

Other researchers have also investigated the chemical composition of *L. salicaria* L. extracts. To the best of our knowledge, this is the first time that the presence of resveratrol in *Lythrum salicaria* L. extracts has been reported. Resveratrol is a phenolic compound with therapeutic effects in cancer, cardiovascular, respiratory, and age-related disorders [44].

Bencsik et al. (2013) reported a comparable amount of gallic acid (166.3 μg/g) and a lower concentration of rutin (1.1 μg/g) in a hydro-ethanolic (50% ethanol) extract from *Lythri herba* collected from Hungary [5]. According to a review article published by Kahkeshani et al. (2019), besides its antioxidant properties, gallic acid protects against inflammation, diabetes mellitus and lipid metabolism disorders [45]. Rutin has antioxidant, antiapoptotic, and anti-inflammatory effects, which are involved in combating liver, kidney, and heart toxicity induced by other substances [46]. Turker et al. (2021) reported that a methanolic extract of *L. salicaria* L. collected from Turkey contained a higher concentration of gallic acid (1.24 mg/g dry extract) [6]. Srećković et al. (2020) also reported that an aerial part extract of *L. salicaria* L. collected from Serbia contained a greater amount of gallic acid (2.10 mg/g dry extract) [3]. Safta et al. (2025) exhibited that an optimized extract of *L. salicaria* L. collected from Cluj County, Romania, contains the following main phenolic compounds: gallic acid (29.366 μg/mL), isoquercitrin (10.830 μg/mL), and chlorogenic acid (3.422 μg/mL) [14].

Varga et al. (2020) investigated the composition of *L. salicaria* L. aerial parts collected from the spontaneous flora of Mureș County, Romania. Their 70% methanolic extract had lower quercetin content (12.79 μg/g) than the extract analyzed in the present study [7]. Quercetin, an important dietary component, is known for its antioxidant, anti-inflammatory, and neuroprotective effects. In addition, clinical studies have highlighted its anticancer, antianemic, and antihypertensive effects, as well as its safety [47,48].

Previous studies reported that *L. salicaria* L. extracts contained caffeic acid [5,6,7], chlorogenic acid [5,7], and catechin [5], which were not determined in our extracts. Differences in bioactive compound concentrations could be explained by variations in plant harvesting time and area, pedoclimatic conditions, types of solvents and methods used for extraction, and the conditions and time of preservation of the vegetal raw material until extraction. Variations in the composition of extracts will inevitably affect their biological properties.

### 3.2. Polyphenol Content and Antioxidant Activity

Table 2 presents the TPC and antioxidant activity of *L. salicaria* L. extracts. The TPC was significantly higher in the LSmet-1 hydro-methanolic extract compared to the LSeth-2 hydro-ethanolic extract. The percentage of DPPH radical scavenging activity was similar for both extracts. The FRAP assay showed that the hydro-methanolic extract exhibited greater antioxidant capacity compared to the hydro-ethanolic one, but the mean values were not significantly different. The ABTS radical cation scavenging assay revealed that the hydro-ethanolic extract had a higher antioxidant activity than the hydro-methanolic extract, although the difference was not statistically significant. Ascorbic acid used as the positive control demonstrated significantly greater antioxidant activity compared to the studied extracts in DPPH, FRAP, and ABTS assays.

The TPC was relatively higher in the hydro-methanolic extract than in the hydro-ethanolic extract because polyphenols are more soluble in methanol than in ethanol. Several studies have reported that polyphenol extraction using methanol as a solvent leads to a higher yield compared to ethanol [49,50]. Moreover, combining methanol or ethanol with water in various ratios can enhance the polyphenol content of the extracts [50]. Tunalier et al. (2007) reported a higher total phenolic content in *L. salicaria* L. hydro-methanolic extract (525.76 mg GAE/g dry weight) compared to methanolic extract (191.35 mg GAE/g dry weight) and water extract (305.22 mg GAE/g dry weight) [8].

Some authors suggest that a concentration above 20 mg GAE/g indicates that an extract could be considered rich in phenolic compounds; however, there is no universally established threshold beyond which the phenolic content is considered high [51]. Compared to our results, Mohammadalinejhad et al. (2019) reported that 80% hydro-methanolic extract from the flowering aerial parts of *L. salicaria* L. have a similar total phenolic content of 23.03 mg GAE/g dry weight [52]. Kähkönen et al. (1999) reported a higher total phenolic content of 42.1 mg GAE/g dry weight [51]. Manayi et al. (2013) and Vafi et al. (2016) reported a total phenolic content of 331 μg GAE/mg in a hydro-methanolic extract of *L. salicaria* L. aerial parts [10,18]. Turker et al. (2021) reported a total phenolic content of 311.88 mg GAE/g of dry extract [6], while Lopes et al. (2016) found that an 80% acetone extract of *L. salicaria* L. had a total phenolic content of 278 mg GAE/g dry weight [12].

The composition of the extracts varies depending on the collection areas and the methods used to extract the material [10]. Polyphenol content also depends on the specific plant organ and the harvesting period. Bencsik et al. (2011) reported that the flowering parts of *L. salicaria* L. contain higher amounts of polyphenols compared to the leaves and shoots. They also observed that the polyphenol content in the flowering parts is higher in August than in July, while the content in leaves and shoots is higher in July than in August [53]. Srećković et al. (2021) reported that the total phenolic content of *L. salicaria* L. aqueous extract from aerial parts was 99.56 mg GAE/g dry plant and 26.44 mg GAE/g dry plant for *L. salicaria* L. aqueous extract from roots [13]. Conversely, in another study, Srećković et al. (2020) found that root extracts had a higher polyphenol content (326.36 mg GAE/g dry weight) compared to aerial part extracts (201.5 mg GAE/g dry weight) [3]. Engin et al. (2022) incorporated *L. salicaria* L. extract into sodium alginate films and observed that the total phenolic content of the films varied between 15.47 and 69.47 mg GAE/g film, depending on the concentration of the extract [32].

Although the LSeth-2 extract had a lower content of phenolic compounds, its antioxidant activity was not significantly different from that of the LSmet-1 extract. This suggests that other substances from the extracts (for example, ascorbic acid) could exert an antioxidant effect. Nevertheless, Tunalier et al. (2007) observed a stronger DPPH scavenging effect for hydro-methanolic extract compared to methanolic and water extracts, as well as a moderate correlation between TPC and DPPH scavenging activity [8]. Compared to our results, similar DPPH inhibition percentages were reported by Iancu et al. (2021) for a water extract of *L. salicaria* L. at a concentration of 2.5 mg/mL (94.39%) [21] and Turker et al. (2021) reported them for a methanolic extract at 200 μg/mL (96.2%) [6]. Pirvu et al. (2014) found that *L. salicaria* L. extract had a higher DPPH scavenging potential compared to rutin and gallic acid used as standards [9]. Manayi et al. (2013) observed that DPPH scavenging activity was higher for the 80% methanolic extract than for vitamin E, attributing the antioxidant effect to its phenolic content [10]. López et al. (2008) demonstrated that the methanolic extract of *L. salicaria* L. had a stronger DPPH scavenging effect than butylhydroxytoluene (BHT) and a comparable effect to ascorbic acid [11]. Lopes et al. (2016) reported high antioxidant activity for the 80% acetone extract of *L. salicaria* L., as measured by DPPH and ABTS scavenging assays [12]. Compared to our results, Mantle et al. (2000) found that 80% hydro-ethanolic leaf extract exhibited a relatively lower antioxidant activity of 0.31 mmol TE/g dry weight when tested by using the ABTS method [54]. Safta et al. (2025) optimized extraction conditions in order to obtain an extract with a high content of polyphenols and flavonoids that are associated with antioxidant capacity, demonstrated by DPPH, FRAP, and trolox equivalent antioxidant capacity (TEAC) assays [14]. Therefore, it is likely that variations in antioxidant activity are also due to differences in extract composition.

According to Tian et al. (2021), the antioxidant capacity of flavonoids is positively correlated with the number of hydroxyl groups from their chemical structure; thus, the high antioxidant potential determined for the extracts analyzed may be due to the high concentration of quercetin, the compound with the most hydroxyl groups among the other flavonoids analyzed [55]. The lack of significant differences in the antioxidant activities of the tested LSmet-1 and LSeth-2 extracts suggests that the hydro-ethanolic extract could safely replace methanol in pharmaceutical products, avoiding its toxicity without compromising the therapeutic effect.

### 3.3. In Vivo Drosophila melanogaster Viability Tests on Neutral and Normal Diets

When considering a plant extract for therapeutic use, its potential toxicity should be evaluated using an in vivo model system in a concentration-dependent manner. In this regard, we have developed a system based on fruit flies and their diet that is suitable for detecting position-effect variegation (PEV). We can analyze the influence of specific nutrition on the biological effects of a given plant extract by applying three types of diets to individuals with identical genotypes and epigenomes. Furthermore, all the tested *Drosophila* individuals are the same age. By assessing concentration dependency and the timing of their life cycles, information could be generated regarding the possible correlation between the concentration of plant extracts and the life cycle development of *Drosophila*. This fact provides an outstanding competitive advantage, given that the outcome of human trials is greatly influenced by genetic variability and the age of individuals. Therefore, the *Drosophila*-based viability test provides basic information that could be used to design more targeted human trials.

In the case of the neutral diet that provides no nutrients to newly hatched *Drosophila* larvae, due to the absence of nutrients, they will die instantly. However, if the neutral diet is supplemented with plant extracts containing macro- and/or micronutrients, then the nutritional effects of the corresponding extracts can be tested in vitro by monitoring the life cycle of individuals. At 25 °C, 0–2-hour-old embryos will develop into first-instar larvae within 24 h. With a normal diet, they will reach the second-instar larval stage within an additional 24 h. After one more day, they will reach the third-instar larval stage. In three more days, they will reach the pupal stage, and metamorphosis will last another four to five days, followed by the hatching of adults. However, if the plant extract being tested is unable to provide the necessary nutrients, the life cycle will stop, and the individuals will fail to reach the next stage of life. If the plant extract being tested contains the necessary nutrients, and if individuals reach adulthood by assessing the eye pigment content of two- to four-day-old male individuals, then the expression level of the white gene can be inferred. The tested Drosophila *w^m4h^* strain has the *w* gene repositioned from the distal end of the X chromosome, an euchromatic region, to the vicinity of the pericentric heterochromatin that can cause a variegated *w* phenotype. The level of variegation or the expression level of the repositioned *w* gene depends on the spread over or blocking of heterochromatinization over the *w* gene [56,57]. Experimental evidence suggests that background mutations are the major contributors to this variegation [58], but other factors that might interfere with chromatin organization could possibly affect variegation and gene expression [59].

On a neutral medium, larvae were observed on day 2 at a concentration of 4.76% LSmet-3, with day 0 considered the seeding day. By day 10, some larvae died, and by day 15, most were dead. By day 19, all the larvae died. This suggests that the LSmet-3 extract contained some nutrients but not enough to support the larva and pupal stages of the life cycle. At a concentration of 20% LSmet-3, first- and second-instar larvae were visible on day 3, with one pupa observed on day 12. At a concentration of 33.33% LSmet-3, first- and second-instar larvae were visible on day 6. No larvae were detected from the second day onwards at a concentration of 50% LSmet-3. It is therefore likely that the increasing LSmet-3 concentration in the neutral medium did not fully support the viability of *Drosophila* larval/pupal development.

**Figure 1:**
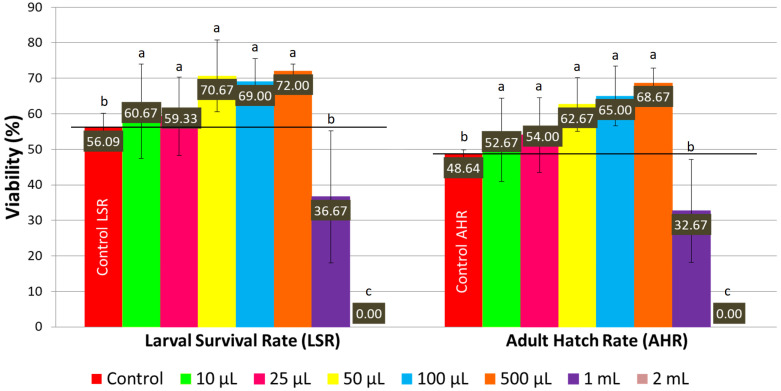
The larval survival rate and adult hatch rate with a normal diet. Different letters (a, b, c) indicate a significant difference between the means (*p* < 0.05) and error bars represent the standard deviation.

**Figure 2:**
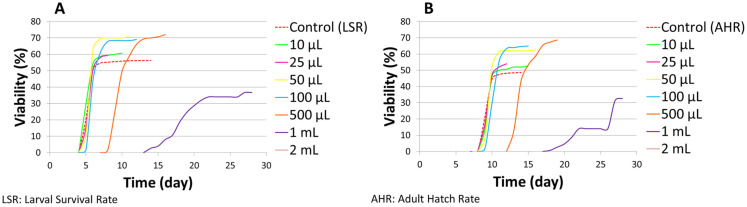
Larval survival rate (**A**) and adult hatch rate (**B**) curve on normal medium.

On a normal medium, concentrations of LSmet-3 extract ranging from 10 to 500 μL per 2 mL of normal medium significantly increased the larval viability of *Drosophila melanogaster* compared to the control (see Figure 1 and Figure 2). The larval viability increased proportionally to the concentration of the LSmet-3 extract. As expected, the highest adult hatch rate (68.67%) was observed at a concentration of 500 μL of LSmet-3 extract per 2 mL of normal medium compared to an adult hatch rate of 48.64% in the control group. Furthermore, the concentrations of 1 mL and 2 mL of LSmet-3 extract in 2 mL of normal medium were assessed. The 1 mL concentration was found to significantly decrease adult viability, while the second 2 mL concentration appeared completely toxic, resulting in no hatched adult individuals.

In addition to viability, the developmental timing of the LSmet-3 extract/normal-media-fed individuals was monitored. Clearly, there were no differences with respect to controls in the 10–100 µL concentration range (see Figure 2). However, a clear time shift could be observed at the 0.5–1 mL concentration range of LSmet-3. Specifically, the larval period was extended by three to eight days compared to the control group. The viability of the treated individuals remained unaffected at a concentration of 500 µL of LSmet-3. However, at a concentration of 1 mL of LSmet-3 in normal media, the viability decreased significantly to 40% compared to the control, which occurred in addition to the relevant developmental shift.

These experiments demonstrated that supplementing a normal diet with LSmet-3 could have detrimental effects at high concentrations. This toxicity has variable strength, initially being associated with delayed larval and pupal development, though it does not affect viability. Ultimately, the further increasing concentrations of LSmet-3 induce a more pronounced developmental shift and, finally, complete lethality at the highest concentration.

### 3.4. The Relevance of the Hormetic Effect of LS Extracts

An assessment of the effect of LSmet-3 extract concentration on the viability of *Drosophila* larvae and adults reveals a clear biphasic dose response with a lower dose stimulatory or beneficial effect and a higher dose inhibitory or toxic effect [60]. The hormetic effect is considered an adaptive, beneficial mechanism generated by a substance or matrix at low doses but toxic at higher concentrations [61].

Explaining the biphasic effect of the LSmet-3 extract is both challenging and puzzling. While the experiment is reproducible and the concentration dependence on the effects is evident, the relevance of the extract composition is worth considering.

Previous research conducted by Ogunsuyi et al. (2020) had found no significant changes in survival rates of *Drosophila melanogaster* that were fed with 50 and 100 μM of gallic acid. In the same study, gallic acid demonstrated antioxidant effect by improving oxidative stress parameters such as ROS, malondialdehyde, catalase, and total thiol in a *Drosophila melanogaster* model of Alzheimer’s disease [62].

Another study demonstrated that rutin exerts a hormetic effect that is responsible for lifespan extension in fruit flies. They reported that 200 and 400 μM of rutin significantly increased the median lifespan of *Drosophila melanogaster*. At 100 μM, rutin did not change the median survival compared to the control, while concentrations of 600 and 800 μM significantly decreased the median survival rate [63]. Since the LSmet-1-derived LSmet-3 and LSmet-2 extracts contain rutin, a phytonutrient, they are expected to feature a hormetic effect as demonstrated by our experiments with LSmet-3.

Regarding resveratrol, another component of the LS extract, other researchers observed that supplementing food with 200 μM resveratrol significantly increased the viability of larval and adult fruit flies compared to the control group. Additionally, no significant differences were reported for both lower (25, 50, and 100 μM) and higher (800 μM) concentrations, indicating that resveratrol could also generate a hormetic effect [64].

In another study, the dose dependency of quercetin was monitored in *Drosophila melanogaster* [65]. It was demonstrated that the survival rate of adult individuals decreased in a concentration-dependent manner, though the entire life cycle was not assessed. Nevertheless, when catalase, malondialdehyde, and superoxide dismutase levels were examined, the diphasic nature of these effects were evident. These results suggest that quercetin, a phytonutrient found in the LS extract, could also generate a hormetic-type effect like that of gallic acid, rutin and resveratrol.

Interestingly, when the effects of resveratrol on *Drosophila melanogaster* were assessed again, it was demonstrated that, besides being concentration-dependent, up to 200 µM of resveratrol would not affect the lifespan of wild-type flies when fed a normal diet or a restricted or high-sugar and low-protein diet [66]. However, when resveratrol was administered at 400 µM, the lifespan extended in the case of females on a high-fat diet. This prolongevity effect was mediated through the downregulation of genes implicated in antioxidant responses and insulin signaling.

Another study by Iancu et al. (2021) investigated the water and alcohol extracts of *L. salicaria* L. on *Artemia salina*. They concluded that the tested extracts, which contained polyphenols, tannins, and anthocyanins, were only toxic at relatively high concentrations. They did not assess a wider concentration range, and therefore, based on the obtained results, they considered the extracts non-toxic, a fact that supports LS extract’s potential use as a natural therapeutic alternative [21]. Furthermore, Puisais and Cock (2025) reported that 1 mg/mL LS extracts prepared with different polar and non-polar solvents did not significantly influence the mortality of *Artemia nauplii* compared to the negative control. Furthermore, no concentration range was tested for any of the generated extracts, and as a consequence, no significant differences were observed in mortality [67]. Even though its ability to reduce inflammatory cytokines has been properly demonstrated, the lack of toxicity is insufficient to support the therapeutic use of *Lythrum salicaria* L. when considering the above-mentioned experimental circumstances. Remarkably, Lopes et al. (2016) reported a moderate toxicity of *L. salicaria* L. extract against several in vitro cultured cell lines, including human hepatocarcinoma (HePG2), human cervical adenocarcinoma (HeLa), human leukemia monocytic (THP1), and murine bone marrow stromal (S17) cells [12]. Srećković et al. (2020) also observed a low cytotoxicity of the extract against normal and cancerous cell lines [3]. Safta et al. (2025) observed a dose-dependent increase in toxicity against HaCaT, BJ, and RAW 264.7 cell lines when tested with different concentrations of *L. salicaria* L. optimized extract [14].

There is ample and mostly in vitro experimental evidence pointing towards the dose-dependent toxicity of various LS extracts, in addition to their beneficial physiological effects. However, the biphasic nature of the LS extracts requires further analysis. In this respect, in addition to the extract composition, the diet administered to the tested individuals could interfere with the expected beneficial effect.

### 3.5. In Vivo Drosophila melanogaster Viability Tests with High-Sugar Diet

**Figure 3:**
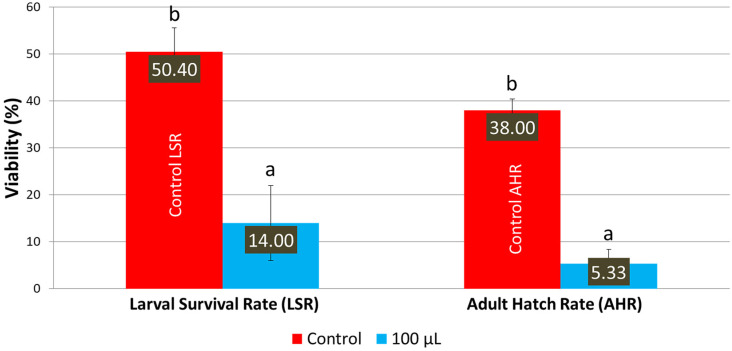
Larval survival and adult hatching rates with the HS diet. Different letters (a and b) indicate a significant difference between the means (*p* < 0.05) and error bars represent the standard deviation.

When the LSmet-3 extract was assessed on an HS diet, the toxic effect of the extract became more pronounced. In the case of a normal diet, the LSmet-3 extract showed increased viability within the 10–500 μL concentration range (see Figure 1). Furthermore, the larval viability was 36.67%, and adult survival was 32.67% at 1 mL. However, it became completely toxic at 2 mL. When a similar experiment was performed and the normal diet was replaced with an HS one, the total toxic effect was visible at a lower LSmet-3 concentration, namely at 500 μL (Figure 3). With the HS diet, larval viability was around 50.40% and the adult survival rate at about 38%. However, when these experiments were performed by testing different concentrations of the LSmet-3 extract in combination with the HS diet, it was found that the survival rates of the larvae and adults dropped to 14% and 5.33%, respectively.

**Figure 4:**
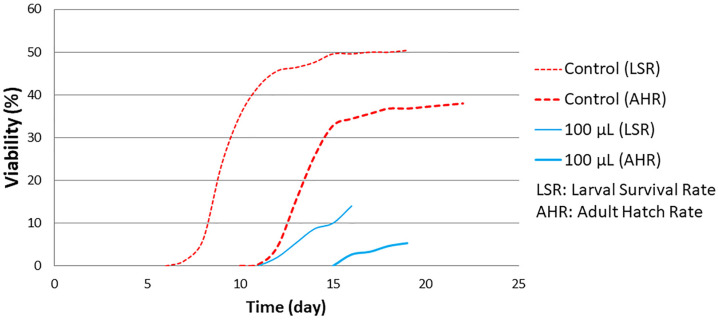
Developmental timing of larval survival and adult hatch rate curve with HS diet.

The results obtained suggest that the HS diet exacerbates the toxic effects of the LSmet-3 extract. At a concentration of 500 μL of the extract, no viable third-instar larvae or adults were detected. This suggests that the interaction between the HS diet and the LSmet-3 extract increases the level of toxicity. Nevertheless, this toxicity also features a developmental shift that becomes more pronounced at the concentration of 100 μL of LSmet-3 (see Figure 4).

This developmental shift takes about five days, but almost all larvae entering the pupal stage complete metamorphosis, suggesting that toxicity is mostly linked to larval development-specific events.

It has been reported that a high-sugar (HS) diet negatively impacts the development of *Drosophila melanogaster*. Musselman et al. (2011) demonstrated that a diet high in sugar promoted diabetes and obesity in fruit flies, while hyperglycemia, insulin resistance, and fat accumulation were also observed. They highlighted that gene expression changes induced by a high-sugar diet in flies were like those involved in the development of type 2 diabetes in humans [68]. Ecker et al. (2017) demonstrated that a high-sugar diet delayed larval/pupal development and reduced pupal viability compared to a normal diet. They also reported that the mentioned diet affected insulin-responsive genes and induced oxidative stress [69]. Thus, the low viability in the case of the high-sugar diet could be caused by metabolic disturbances and oxidative stress, which make *Drosophila melanogaster* more susceptible to the toxic action of the LSmet-3 extract. Moreover, it has been demonstrated that plant extract-generated physiological effects could also be influenced by an HS diet like in the case of olive, sweet almond and black mulberry gemmotherapy extracts (GTEs) compared to those with a normal diet [42]. The GTEs all had a two-phase effect on developmental delay under the HS diet. The viability of sweet almonds remained largely unaffected, while the viability of olives and black mulberries increased. Interestingly, the concentration-dependent viability of black mulberry GTE was less pronounced in an HS diet than in a normal diet.

These experiments tell us that special attention should be paid to any plant extract intended for therapeutic use since multiple interactions could emerge, and the concentration dependency of the interacting partners deserves a detailed evaluation.

### 3.6. Modulation of w^m4h^ Gene Expression of Drosophila with LS Extract

The *Drosophila melanogaster w^m4h^* strain has been used to study position-effect variegation (PEV), a phenomenon that refers to the mosaic expression of a *w^m4h^* allele that has been relocated from an euchromatic into a heterochromatic region. Because of this chromosomal rearrangement, the juxtaposition can cause *w^m4h^* allele expression to become suppressed in some eye cells due to the overspread of a heterochromatin type of chromosomal organization, while in other cells, its expression might become enhanced [70]. Quite remarkably, the PEV of *Drosophila* has been used to study heterochromatin formation and the consequent gene silencing, reducing the expression of the *w^m4h^* allele. About 150 genes have currently been identified that function with the onset and/or maintenance of heterochromatic gene silencing and methylation in PEV. In a broader sense, the organization and maintenance of heterochromatin is related to epigenetic functions and/or mechanisms specific to all metazoans [58].

All the toxicological evaluations of the LSmet-3 extract based on *Drosophila melanogaster* were performed on the *w^m4h^* strain. This allowed us to evaluate different viabilities and the eventual chromatin modulatory consequences by monitoring the expression levels of the *w^m4h^* allele and determining the concentration of drosopterins from the eye of adult individuals (see Material and Methods). The *w^m4h^* allele is a variant of the wild-type *white* (*w*) gene that is implicated in the production and distribution of drosopterin pigments found in the compound eyes and ocelli of adult fruit flies [71].

**Figure 5:**
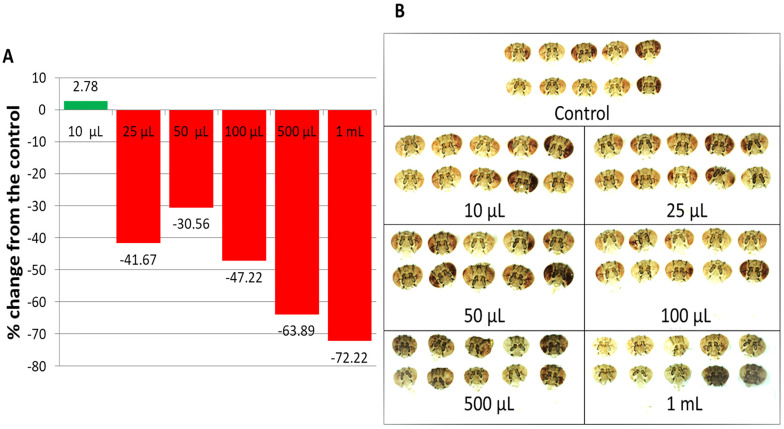
Drosopterin levels in the eyes of *w^m4h^ Drosophila melanogaster* raised on a normal diet supplemented with *Lythrum salicaria* L. extract; (**A**) the drosopterin percentage change from the control; (**B**) images of adult male heads used for the extraction of the ocular pigment (drosopterin). The flies in the lowest concentration group present higher eye pigmentation than the control group. At higher concentrations, the eyes are less colored compared to those of the control group.

Compared to the untreated control, 10 μL of LSmet-3 extract in 2 mL of normal media increased drosopterin levels in the eyes of *w^m4h^ Drosophila melanogaster*. The other tested concentrations resulted in decreased drosopterin levels (Figure 5A,B). These results suggest that the expression of the *w* gene responsible for eye pigmentation may be influenced by the LS extract in a concentration-dependent manner. Interestingly, no previous studies have reported a specific plant extract that modulates gene expression in fruit flies. The exposure of *w^m4h^* flies to the LSmet-3 extract was not constant throughout the experiment because it only lasted during the larval period and the pigmentation of adult eyes would have developed several days later. Therefore, it is reasonable to assume that the levels of *w* gene expression induced by LSmet-3 are not immediate physiological consequences but rather the result of chromatin-based modifications, such as heterochromatinization or epigenetic landmarks, which persist throughout generations. This should be factually demonstrated.

## 4. Conclusions

The reported research focuses on the polyphenol content of extracts obtained from *Lythrum salicaria* L. The main phenolic compounds quantified in hydro-methanol extract were gallic acid, resveratrol, and rutin, while hydro-ethanol extract contained mainly quercetin and gallic acid. These compounds are important for the antioxidant properties of the mentioned extracts. Given the limited experimental evidence about the physiological effect generated by the *Lythrum salicaria* L. extracts, the extract featuring the most abundant polyphenol content was further analyzed for the concentration and diet dependence of viability and gene expression.

Low concentrations of the *Lythrum salicaria* L. extract increased the viability of *Drosophila melanogaster* under normal dietary conditions. However, higher extract concentrations at similar dietary conditions were toxic. Clearly, the biphasic nature of the generated viability was evident.

Furthermore, similar experiments performed at HS dietary conditions showed the *Lythrum salicaria* L. extract to become more toxic at even lower concentrations. Moreover, all tested concentrations of *Lythrum salicaria* L. extract influenced the pigment content from *Drosophila melanogaster* eyes, suggesting the possibility of some extract-specific phytoconstituent(s) implication in generating physiological mechanism(s) affecting gene expression. This kind of generated physiological data pleads for a more careful analysis with respect to the possible therapeutic use of *Lythrum salicaria* L. extracts. Ultimately, our study contributes to expanding knowledge on medicinal plants from local regions, enhancing their potential for exploitation in the development of pharmaceutical products.

## Figures and Tables

**Table 1:** Phenolic profile of *Lythrum salicaria* L. extracts.

Phenolic Compound (μg/g d.p.)		Sample	
		LSmet-1	LSeth-2
Phenolic acids	Gallic acid	183.82 ± 1.13	134.83 ± 1.50
	Cinnamic acid	4.58 ± 0.22	23.46 ± 0.53
	Syringic acid	27.30 ± 0.73	31.15 ± 0.67
	Caffeic acid	n.d.	n.d.
	Chlorogenic acid	n.d.	n.d.
	Ferulic acid	9.22 ± 0.24	14.92 ± 0.08
Flavonoids	(+)-Catechin	n.d.	n.d.
	Rutin	68.09 ± 2.14	21.99 ± 0.33
	Quercetin	28.81 ± 0.71	200.75 ± 1.94
Stilbenes	Resveratrol	76.48 ± 1.51	15.06 ± 0.92

LS: *Lythrum salicaria* L. aerial part extract; 1: methanol/water (70:30, *v*/*v*) extract; 2: ethanol/water (70:30, *v*/*v*) extract; d.p.: dry plant; n.d.: not determined. The experiments were performed in triplicate (n = 3) and the results were expressed as the mean ± standard deviation (SD). Values from the same row are significantly different (*p* < 0.05).

**Table 2:** Total polyphenol content and antioxidant activity of *L. salicaria* L. extracts.

	Sample	LSmet-1	LSeth-2	Ascorbic Acid
Assay				
TPC (mg GAE/g d.p.)		26.40 ± 1.42 ^a^	13.38 ± 0.49 ^b^	n.d.
DPPH (%)		93.24 ± 0.07 ^a^	93.04 ± 0.19 ^a^	100.00 ± 0.42 ^b^*
FRAP (µmol TE/g d.p.)		31.04 ± 0.60 ^a^	12.44 ± 0.44 ^a^	20.07 ± 1.09 ^b^ mmol TE/g ascorbic acid
ABTS (mmol TE/g d.p.)		0.37 ± 0.05 ^a^	0.43 ± 0.02 ^a^	21.45 ± 1.53 ^b^ mmol TE/g ascorbic acid

TPC: total polyphenol content; DPPH: DPPH• radical scavenging assay; FRAP: ferric-reducing antioxidant power; ABTS: ABTS•+ radical cation scavenging assay; GAE: gallic acid equivalents; TE: trolox equivalents; LS: *Lythrum salicaria* L. aerial parts extract; 1: methanol/water (70:30, *v*/*v*) extract; 2: ethanol/water (70:30, *v*/*v*) extract; d.p.: dry plant; n.d.: not determined; *: ascorbic acid solution 1 mg/mL. The experiments were performed in triplicate (n = 3) and the results were expressed as the mean ± standard deviation (SD). Values from the same row with different letters are significantly different (*p* < 0.05).

## Data Availability

All data are included in the article. Further inquiries can be directed to the corresponding author.

## Supplementary Materials

The following supporting information can be downloaded at: https://www.mdpi.com/article/10.3390/pharmaceutics17091097/s1, Figure S1. Chemical structures of the analyzed compounds, Figure S2. HPLC Chromatogram of the used standard phenolic compounds in hydro-methanolic solvent, Figure S3. HPLC Chromatogram of the used standard phenolic compounds in hydro-ethanolic solvent, Table S1. Regression equations for the analyzed compounds.
