# Supplementary material for: Assessment of Purple Loosestrife (Lythrum salicaria L.) Extracts from Wild Flora of Transylvania: Phenolic Profile, Antioxidant Activity, In Vivo Toxicity, and Gene Expression Variegation Studies
Source: Pharmaceutics. 2025 Aug 22;17(9):1097. doi: 10.3390/pharmaceutics17091097 (PMC12473393; doi:10.3390/pharmaceutics17091097)
Supplement: Supplementary file 1 [file pharmaceutics-17-01097-s001.zip › pharmaceutics-3801199-supplementary.pdf]

SUPPLEMENTARY MATERIAL

Supplementary Figures

Figure S1. Chemical structures of the analyzed compounds

Figure S2. HPLC Chromatogram of the used standard phenolic compounds in hydro-methanolic solvent

Figure S3. HPLC Chromatogram of the used standard phenolic compounds in hydro-ethanolic solvent

Supplementary Table

Table S1. Regression equations for the analyzed compounds

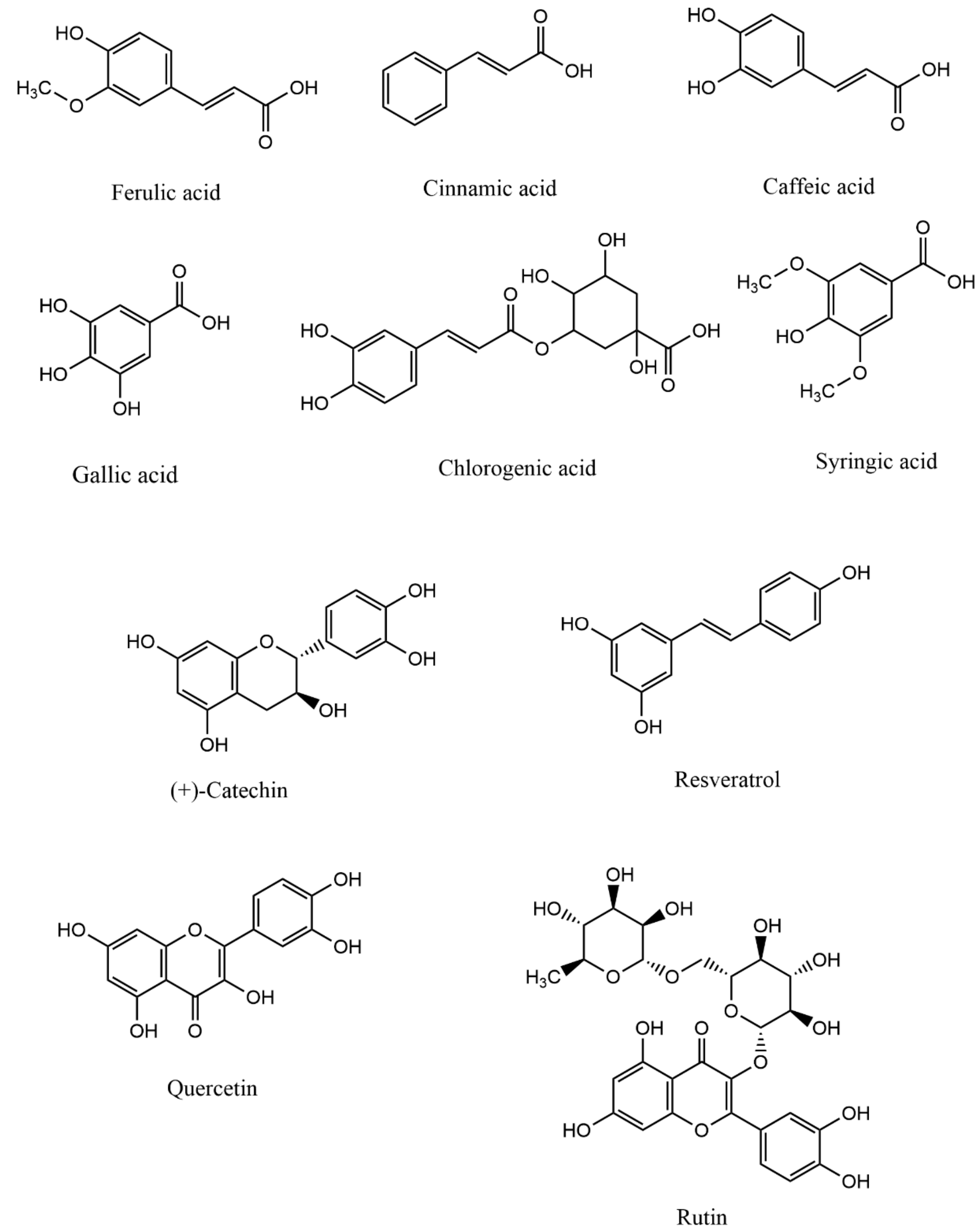

Figure S1. Chemical structures of the analyzed compounds.

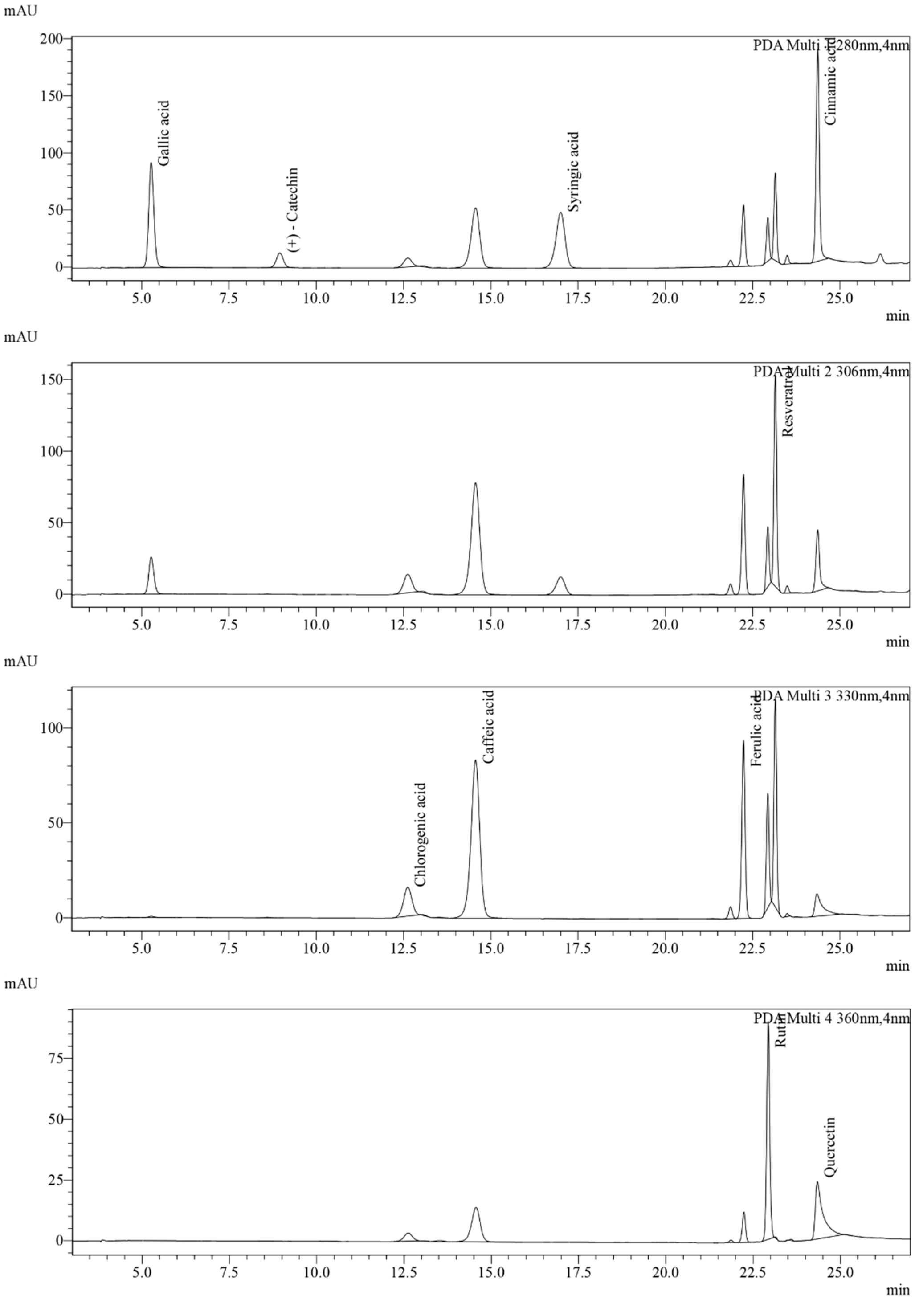

Figure S2. HPLC Chromatogram of the used standard phenolic compounds in hydro-methanolic solvent.

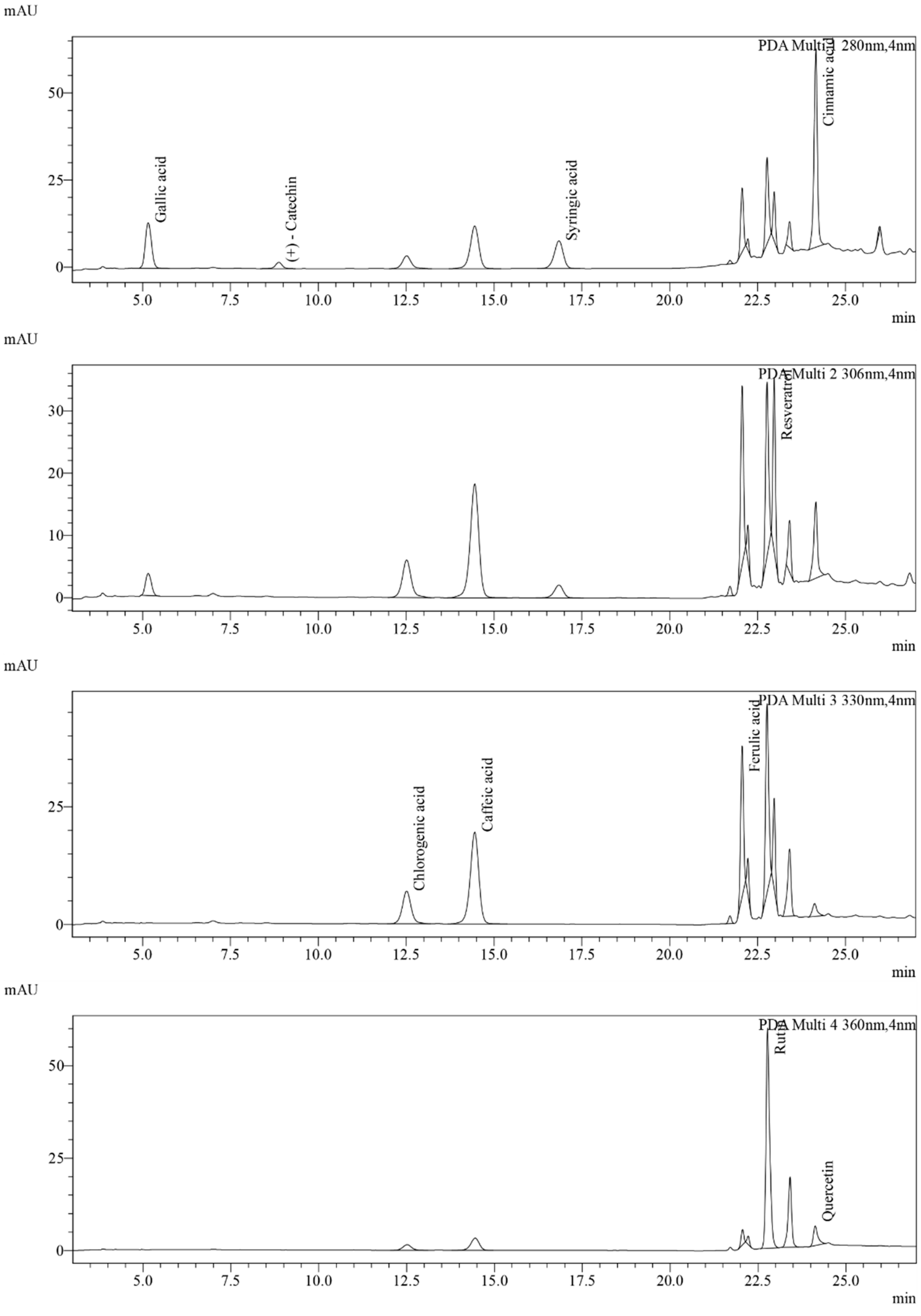

Figure S3. HPLC Chromatogram of the used standard phenolic compounds in hydro-ethanolic solvent.

Table S1. Regression equations for the analyzed compounds.

Chemical compound	Regression equation*	
	**Hydro-methanolic solvent**	**Hydro-ethanolic solvent**
Gallic acid	y = 17559x - 1529.3	y = 11149x + 20456

(+)-Catechin	y = 4073x - 213.8	y = 4558.3x - 1948.4
Syringic acid	y = 18858x - 1382.7	y = 20098x - 6782.7
Cinnamic acid	y = 4073x - 213.8	y = 59866x - 2628.1
Resveratrol	y = 37958x - 23946	y = 47108x - 7729.5
Chlorogenic acid	y = 11519x - 3833.9	y = 16410x - 6168.6
Caffeic acid	y = 11519x - 3833.9	y = 37585x - 11155
Ferulic acid	y = 27612x - 16576	y = 33243x - 17002
Rutin	y = 9926.8x - 423.7	y = 11156x + 257.3
Quercetin	y = 10289x - 11229	y = 22185x - 13525

* Determination coefficient (R^2) > 0.999 for all the compounds analyzed.
